# Feet first: Adaptive growth in magellanic penguin chicks

**DOI:** 10.1002/ece3.7331

**Published:** 2021-03-13

**Authors:** Natasha J. Gownaris, P. Dee Boersma

**Affiliations:** ^1^ Environmental Studies Gettysburg College Gettysburg PA USA; ^2^ Department of Biology University of Washington Seattle WA USA; ^3^ Center for Ecosystem Sentinels University of Washington Seattle WA USA

**Keywords:** adaptive growth, allometry, body condition, canalization, developmental plasticity, energy allocation, natural selection

## Abstract

Growing animals should allocate their limited resources in ways that maximize survival. Seabird chicks must balance the growth of features and fat reserves needed to survive on land with those needed to successfully fledge and survive at sea. We used a large, 34‐year dataset to examine energy allocation in Magellanic penguin chicks. Based on the temporal trends in the selective pressures that chicks faced, we developed predictions relating to the timing of skeletal feature growth (Prediction 1), variation in skeletal feature size and shape (Prediction 2), and responses to periods of high energetic constraint (Prediction 3). We tested our predictions using descriptive statistics, generalized additive models, and principal component analysis. Nearly all of our predictions were supported. Chicks grew their feet first, then their flippers. They continued to grow their bill after fledging (Prediction 1). Variance in feature size increased in young chicks but declined before fledging; this variance was largely driven by overall size rather than by shape (Prediction 2). Chicks that died grew slower and varied more in feature size than those that fledged (Prediction 2). Skeletal features grew rapidly prior to thermoregulation and feet and flippers were 90% grown prior to juvenile feather growth; both thermoregulation and feather growth are energetically expensive (Prediction 3). To avoid starvation, chicks prioritized storing mass during the first 10 days after hatching; then, the body condition of chicks began to decline (Prediction 3). In contrast to our prediction of mass prioritization in young chicks, chicks that were relatively light for their age had high skeletal size to mass ratios. Chicks did not show evidence of reaching physiological growth limits (Prediction 3). By examining energy allocation patterns at fine temporal scales and in the context of detailed natural history data, we provide insight into the trade‐offs faced by growing animals.

## Graphical abstract

Magellanic penguin chicks show adaptive growth. The temporal patterns in their energy allocation are closely linked to their natural history—chicks store mass when the risk of starvation is highest and grow skeletal features in the order that they are needed to survive (feet first, then flippers, and finally bills). By the time that they fledge, chicks vary little in skeletal size or shape.

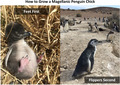

## 1 INTRODUCTION

Research on natural selection theory, in which organisms seek to maximize lifetime fitness, focuses on trade‐offs between current and future reproduction (Stearns, 1989). Less studied is how juvenile animals allocate energy to maximize their chance of reaching reproductive age. Although growing animals often cannot control their limited resource pool, they can allocate resources in response to extrinsic selective pressures (adaptive growth hypothesis; Benowitz‐Fredericks et al., 2006; Cheng & Martin, 2012; O'Connor, 1977). In juvenile mollusks, for example, growth of shell versus soft tissue can be predicted based on species' relative risk of predation prior to and following maturation (Irie & Iwasa, 2004).

Birds provide an interesting case study of adaptive growth because, while mobility is key to survival throughout all life stages, the shift from chicks to adults is often marked by a change in locomotory mode. All birds are relatively constrained in their adult form (e.g., wing‐loading constraints: Sullivan et al., 2019), but their maturity at hatching (i.e., the altricial–precocial spectrum) and their growth speed and trajectory vary (Starck & Ricklefs, 1998). To survive to fledge, chicks must optimize how they allocate energy among the growth of skeletal elements, the maturation of tissues, and the deposition of fat reserves (Starck & Ricklefs, 1998). Here, we used our exceptionally large, long‐term dataset to examine how known‐age Magellanic penguin (*Spheniscus magellanicus*) chicks at Punta Tombo, Argentina, allocate their energy as they grow.

The Magellanic penguin is a long‐lived, serially monogamous seabird with semi‐precocial chicks (Boersma et al., 2013). Both chick survival (35 ± 18% [$\bar{x}$ ± *SD*]; Boersma & Rebstock, 2014) and juvenile survival (12 ± 11% for females, 17 ± 14% for males; Gownaris & Boersma, 2019) are low and show high interannual variability at Punta Tombo. Chicks face a variety of pressures before fledging, including starvation, predation, intruding adult penguins, rainstorms, and extreme heat. Starvation is the greatest cause of mortality in most years (Boersma & Rebstock, 2014; Boersma & Stokes, 1995). When they fledge, juvenile penguins leave Punta Tombo to travel thousands of kilometers north in search of food. They are susceptible to starvation during this period and often travel farther than adults do (Stokes et al., 2014).

A Magellanic penguin chick's access to resources will depend on a range of interacting factors, including hatch order, parental quality and preferences, nest type and cover, and oceanographic conditions (e.g., Barrionuevo et al., 2018; Boersma, 1992; Boersma et al., 2013; Frere et al., 1992; Stokes & Boersma, 1998). Food availability and predictability for penguins breeding at Punta Tombo vary across temporal scales. These penguins forage in tidal fronts along the Patagonian shelf and show a “commuting” foraging pattern and high site fidelity, both of which indicate travel to a predictable food source (Boersma et al., 2009; Weimerskirch, 2007). Tidal fronts in this region coincide with the Magellanic penguin's annual breeding season and are associated with higher concentrations of their main diet items: anchovy, hake, and squid (Acha et al., 2004). Although this food source is spatially predictable, adult foraging distance and reproductive success are highly variable year‐to‐year at Punta Tombo, indicating that food availability varies interannually (Boersma, 2008; Boersma & Rebstock, 2009, 2014; Boersma et al., 2009). Rates of chick starvation also show clear temporal patterns within a season, with mortality peaking in the first 10 days after a chick hatches and again between 40 and 60 days of age, indicating that resource constraints are highest during these periods (Boersma & Stokes, 1995).

How much, how often, and what a chick is fed influences how it allocates its energy (Boersma, 1986; Boersma & Parrish, 1998; Ricklefs et al., 1987). When resources are limited or unpredictable, avian chicks show a variety of responses. Some species prioritize fat reserves (e.g., Alpine swifts *Apus melba*: Bize et al., 2003), some prioritize skeletal feature growth (e.g., California gull *Larus californicus*: Carrier & Leon, 1990, Elegant Terns *Sterna elegans*: Dahdul & Horn, 2003), and others arrest growth (e.g., white‐fronted bee‐eaters *Merops bullockoides*: Emlen et al., 1991; alcids: Kitaysky, 1999) or become hypothermic (Fork‐tailed Storm‐Petrel *Oceanodroma furcata*: Boersma, 1986). When resources are limiting, some skeletal features may be prioritized over others, and which features are prioritized may change as a chick grows (Huxley, 1932; Klingenberg, 2016; Pélabon et al., 2013; Thompson, 1917). Prioritization among skeletal features can be considered through two lenses: (a) the average growth trajectory: the relative size of features at hatching and the order in which features grow to full size, (b) the degree to which growth of a feature is maintained when resources are scarce.

Because survival prior to and immediately following fledging is an important bottleneck in many seabird species, selection during these stages may influence adult form (i.e., ontogenetic inertia; Gignac & Santana, 2016; in crocodiles: Gignac & O’Brien, 2016; in lizards: Herrel et al., 2016). When there is strong selection on chicks and juveniles, some phenotypes may be lost completely from the population prior to reproductive age (the "invisible fraction"; Grafen, 1988). In Magellanic penguins, the size of morphological traits (foot length, flipper length, bill length, bill depth) is heritable and the probability of returning to the colony is higher for individuals with larger feet and flippers (Koehn et al., 2016). Bill size at fledging, however, is similar among chicks that return to the colony and those that do not (Koehn et al., 2016). The influence of adult morphology on reproductive success varies interannually in both strength and direction and may relate to food availability (Koehn et al., 2016).

**FIGURE 1:**
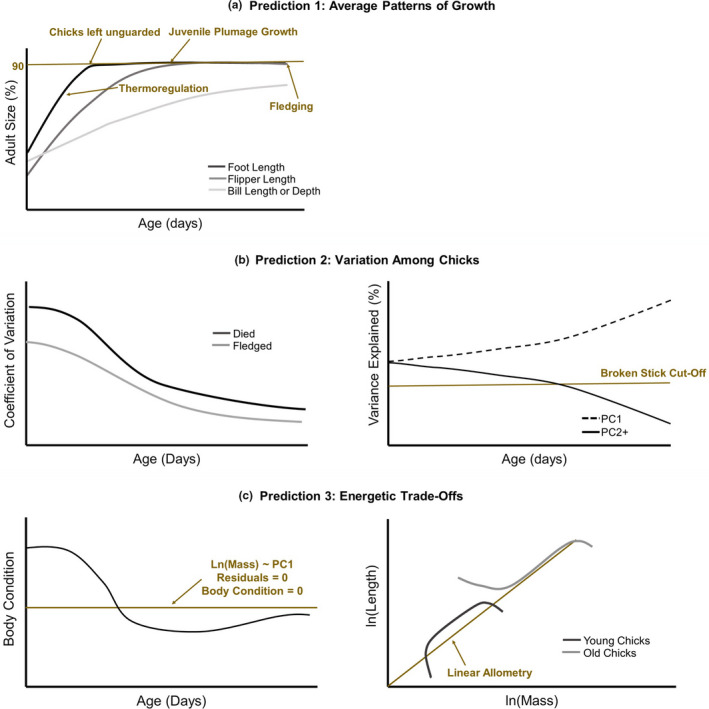
We tested three sets of predictions on adaptive growth in Magellanic penguin chicks at Punta Tombo, Argentina. To test these predictions, we used age‐specific data on growth for 9,491 known‐aged chicks hatched from 1983 to 2017. We predicted (Prediction 1) that the average growth pattern of chicks would be shaped by temporal patterns in the selective pressures they faced and that chicks would grow their feet first, their flippers second, and their bills last (a). To test Prediction 1, we compared the size of features at hatching and the time it took features to reaching 90% of the adult size (a). We predicted (Prediction 2) that variation in the size of these skeletal features would decline over time and that chicks would converge on a similar shape prior to fledging (b). To test Prediction 2, we examined age‐driven trends in the coefficient of variation of individual features and compared these trends between chicks that eventually died and those that eventually fledged (b). We also examined age‐driven trends in the variation described by the first principal component (PC1; “overall size”) of a principal component analysis on all skeletal features, with higher‐order components (PC2+) representing variation in “shape” (b). We predicted (Prediction 3) that young chicks would prioritize mass over skeletal size and that older chicks would prioritize skeletal size over mass (c). To test Prediction 3, we examined age‐driven trends in chick body condition using the residuals of a regression on log‐transformed mass and PC1 and variation in age‐specific allometry using generalized additive models (c)

Our study focuses on how natural selection shapes growth patterns in Magellanic penguin chicks prior to fledging. Using the predictions outlined below and visualized in Figure 1, we link these patterns to the selective pressures that chicks face as they grow and ask whether and why chicks deviate from this average growth pattern.

1. Prediction 1: Average Patterns of Growth. We predicted that, to avoid starvation and predation on land, chicks should grow their feet first. Fledglings will die of starvation if they cannot swim efficiently and capture prey, so we predicted that flippers would be the second feature to reach adult size. While in the nest, chicks use their bills as a funnel to receive food from parents, but once they leave to forage in the ocean, longer or thicker bill at fledging could allow for more diverse prey capture (Holmes & Pitelka, 1968; Hulsman, 1981). Unlike feet and flippers, however, bill size is not a predictor of whether a Magellanic penguin fledgling survives (Koehn et al., 2016); we therefore predicted that bill length and bill depth will be the last skeletal features to reach adult size.
2. Prediction 2: Variation Among Chicks. Due to variation in parental quality and other factors, chicks of the same age may vary substantially in size. If growth rates of some but not other skeletal features are resource‐dependent, chicks should vary in both shape and overall size. However, chicks should converge on a similar size and shape before fledging due to (a) loss of some phenotypes from the population due to selective mortality (Fisher, 1930), (b) canalization of a particular shape to minimize drag, reduce heat loss, and efficiently capture prey at sea (Bookstein & Mitteroecker, 2014; Zelditch et al., 1993). We predicted that variation in the size of skeletal features and shape of chicks would decrease throughout the chick period and that this decline would be steepest in young chicks, which experience the highest mortality rates. We also predicted that this variation would be greater among chicks that eventually died than among chicks that eventually fledged.
3. Prediction 3: Energetic Trade‐Offs. Chicks should grow rapidly in mass and in skeletal size prior to the start of the energy‐intensive processes of thermoregulation and feather growth. This prediction is based on the energy allocation hypothesis, which suggests that energy‐intensive processes should be separated in time because of limits on the amount of energy that parents can provide to their chicks (Węgrzyn, 2013). Based on patterns of chick starvation, chicks are likely to be most energetically limited during the first 10 days posthatching and, to a lesser extent, at 40 to 60 days of age (Boersma & Stokes, 1995). In some species of seabird (e.g., yellow‐eyed penguins *Megadyptes antipodes*: van Heezik & Davis, 1990), resource‐limited chicks prioritize continued growth of skeletal elements, while in other species (Caspian terns *Hydroprogne caspia*: Lyons & Roby, 2011) allometric relationships are maintained even when resources are limiting. We predicted that Magellanic penguin chicks would prioritize mass over skeletal growth during the first 10 days after hatching and would then prioritize skeletal growth. We expected these patterns to be apparent at two temporal scales, across ages (chicks will rapidly grow in mass and body condition when young and skeletal size when older) and among chicks of a given age (light chicks of a given age will prioritize mass when young and skeletal size when older). We expected skeletal feature size to be most closely associated with mass in young chicks, when starvation rates are highest and therefore resources are most limiting.

## 2 METHODS

Magellanic penguins nest in bushes or burrows, provide biparental care, and generally lay two eggs each year. Eggs are usually laid in October and hatch in November or early December after a 40‐day incubation period (Boersma & Rebstock, 2011; Boersma et al., 1990). The first chick hatches an average of 2 days before the second chick and is more likely to survive in most years (Boersma, 1992; Boersma & Stokes, 1995). If both chicks survive to 20 days, parents feed them similar amounts (Wagner & Boersma, 2019). Chicks fledge in January or February by walking from their nest to the sea (Boersma et al., 1990). Egg laying has become later and less synchronous from 1982 to 2019, but there has been no change in the timing of fledging (Boersma & Rebstock, 2014; Cappello and Boersma, Personal communication; Rebstock & Boersma, 2018).

### 2.1 Pressures faced by growing chicks

Like all juvenile animals, Magellanic penguin chicks face a changing landscape of pressures and constraints as they grow. We describe these pressures based on previously published research and on information collected on the behavioral and physiological state of chicks throughout the long‐term study at Punta Tombo (1983–2017). Using this information, chick growth can be categorized into four behavioral stages:

1. Brood—Chicks are continually brooded, or covered by a parent, during the first few days after hatching; parents take turns foraging when chicks are small so that one parent is always at the nest (Boersma & Rebstock, 2009). Chicks cannot thermoregulate for the first 10–15 days of life and being brooded reduces chicks' thermoregulatory cost (Boersma & Rebstock, 2014). Brooding also protects chicks from predators, most commonly Kelp gulls (*Larus dominicanus*) and Antarctic skuas (*Stercorarius antarcticus antarcticus*; Boersma & Rebstock, 2014). The length of time that a chick is brooded likely depends on whether they have been fed, their size (some chicks are too large to be fully brooded by age 5 days; Boersma & Rebstock, 2014), and the ambient temperature. We found that the proportion of brooded chicks was highest during the first week after hatching, then dropped rapidly from 49.9% of chicks 9 days of age to 6.9% of chicks 20 days of age.
2. Guard—Parents continue to trade off on foraging trips and to guard their chicks for 2–3 weeks after the brood stage; the guard period lasts an average of 29 ± 4 days at Punta Tombo (Boersma et al., 2013). Chicks through this stage are fed every 1–3 days (Boersma & Stokes, 1995).
3. Nonguard—Older chicks have greater food requirements and storage capacity, so parents begin to forage simultaneously and take longer foraging trips instead of leaving one parent to guard the nest; chicks are fed larger meals every 3–5 days (Boersma & Rebstock, 2009; Boersma et al., 1990). When chicks are no longer guarded, they may wander from their nests to avoid predators or aggression by nonbreeders and will sometimes join nests with other chicks. Chicks of this species never form large crèches in the open as in some other penguin species (Boersma et al., 2013). They rarely travel far. If they cannot get back to their nest quickly, they risk missing a meal; parents do not feed chicks that are not at their nest (Wagner & Boersma, 2019). The percent of chicks found outside of their nest (“wandering”) increased from 2.7% at age 20 days to 17.4% at age 30 days and was greater than 28% for chicks aged 70 days or older.
4. Fledge—Chicks at Punta Tombo fledged at 78 ± 17 days of age, with a mode of 72 days of age. Chicks have fledged as early as 50 days posthatching and as late as 111 days, but these extremes account for very few chicks (less than 1%). To survive, a chick needs to grow juvenile feathers before fledging. Growing feathers is an energetically expensive process (e.g., cost of molt in macaroni penguins *Eudyptes chrysolophus* and rockhopper penguins *Eudyptes chrysocome*: Brown, 1986). Chicks hatch with their primary down, and by approximately 30 days of age have grown their secondary down (Boersma et al., 2013). They begin to lose their secondary down as early as 40 days of age and on average at 56 ± 9 days of age and completely lose this down (i.e., complete juvenile plumage growth) at 75 ± 9 days of age.

### 2.2 Patterns and drivers of mortality

Reproductive success is dependent on foraging trip distance at Punta Tombo and is highly variable among years, driving fluctuations in chick starvation rates (Boersma & Rebstock, 2009, 2014). On average, 65% of all chicks hatched each year die and 39% die of starvation (Boersma & Rebstock, 2014). From 1983 to 2010, other important drivers of mortality were predation (9% of chicks), rain (6% of chicks), and heat (1% of chicks); the remaining chicks die from rare or unknown causes. Mortality due to rain is particularly variable, killing up to 50% of chicks in some years (Boersma & Rebstock, 2014). Heat deaths are difficult to determine and likely account for a larger proportion of mortality than the reported 1% (Boersma & Rebstock, 2014).

Mortality due to starvation, predation, and rain are all highest in young chicks, and chick mortality peaks during the first 10 days after hatching (Boersma & Rebstock, 2014; Boersma & Stokes, 1995). Chicks that are not fed within a few days of hatching fail to develop their digestive tract so will die of starvation even if their egg sac still contains yolk or if they are later fed (Boersma & Rebstock, 2014). A second wave of mortality often occurs at 40–60 days of age if a chick misses a meal or otherwise does not get fed for five or more days (Boersma & Stokes, 1995).

### 2.3 Growth data

From 1983 to 2017, we marked chicks and measured their mass (kg), foot length, flipper length, bill length, and bill depth (in cm) at hatching and every 5–10 days until they died or fledged. Details on field data collection and on our criteria for inclusion of chicks can be found in Appendix S1.

Nearly all our analyses were run separately for each age of growth; age 20 days, for example, combines data for all known‐aged chicks that were measured on the 20th day after they hatched, regardless of their cohort. This approach reduced the occurrence of repeated measures. For example, first‐ and second‐hatched chicks from the same nest rarely hatch on the same day, so are not measured at the same ages (excluding hatching). Magellanic penguins are serially monogamous, however, and some pairs bred in multiple years of the study (Boersma et al., 2013). Due to a low number of repeated measures per pair, we could not include pair as a random effect in our models. Only 800 of the 3,565 unique known pairs (pairs with two known parents) in the study had more than one chick measured at a specific age (excluding hatching) and of those 800 pairs the average number of repeated measures was only 2.14 ± 0.43 chicks per age. To remove repeated measures, we randomly selected one sample per chick age per pair to create our dataset for analysis; chicks with unknown parents were excluded. For analyses conducted across ages, we randomly selected only one set of measurements per chick, as noted where relevant below.

Our criteria led to a dataset of 40,651 measurements of each feature and mass from 9,491 unique known‐aged chicks. Sample size varied across ages and declined from hatching to fledging, with 3,299 chicks measured at hatching (age of 0), over 300 chicks measured at each age up to age 70, and over 50 chicks measured at each age up to age 80. We used R version 3.6.3 (Holding the Windsock) for all statistical analyses, including the function gam in package mgcv (version 1.8‐31; Wood, 2020) for all regression models, and the tidyverse (1.3.0; Wickham et al., 2019) package for data wrangling, summarizing, and visualizing. Raw skeletal feature and mass data were natural log‐transformed prior to all linear and nonlinear regressions (Huxley, 1932).

### 2.4 Prediction 1: Average patterns of growth

We tested our prediction of growth order and timing by comparing the size of features at hatching, by examining the rate of change in these features, and by estimating the time it took the average size of features to reach 90% of the average adult male size (mass of 4.6 kg, flipper length of 15.6 cm, foot length of 12.2 cm, bill length of 5.82 cm, bill depth of 2.48 cm; Boersma et al., 2013; Figure 1a). We used age‐specific averages of feature sizes to determine the age at which chicks reached 90% of adult size and calculated these values separately for chicks that survived to fledge (*n* = 4,115) versus those that eventually died (*n* = 5,376). To meet our fledging criteria, chicks had to have been alive on or after 10 January and weigh at least 1,800 g (Boersma et al., 1990). We did not include measurements taken on dead chicks in our analysis (Appendix S1), but instead examined the patterns of growth in these chicks prior to their death.

We chose 90% because (a) this is a common benchmark in penguin chick growth studies (e.g., Sherley, 2010; Volkman & Trivelpiece, 1980), (b) female chicks will never reach adult male size (Boersma et al., 2013), and (c) if larger chicks are more likely to return to the colony as adults (Koehn et al., 2016; Naef‐Daenzer & Grüebler, 2016), the average size of fledglings will be smaller than that of adults. As compared to female Magellanic penguins at Punta Tombo, male Magellanic penguins are 21.1% heavier and have bills that are 18.0% longer and 17.5% deeper, flippers that are 6.1% longer, and feet that are 6.1% longer (Boersma et al., 2013). Magellanic penguin males do fledge larger than females on average but, because the size range of their features overlaps substantially at fledging, morphological sexing is not reliable.

To test whether using 90% of adult male size as a benchmark influenced our results, we also examined feature averages for chicks that we recaptured, sexed, and remeasured as adults (*n* = 135 males, 39 females). For this analysis, chick measurements were compared to individual‐specific adult measurements. This subset of our dataset is, by definition, biased toward individuals that survived to return to the colony as adults.

### 2.5 Prediction 2: Variation among chicks

We used the coefficient of variation (CV) to quantify age‐driven trends in variation of each skeletal feature and mass across chicks. We compared these values among chicks that fledged and those that died (Figure 1b).

To quantify variation in skeletal shape, we conducted a principal component analysis (PCA) of centered and scaled skeletal feature data (foot length, flipper length, bill length, bill depth) using the prcomp function in R (Figure 1b). This analysis was run separately for chicks of each age. We also ran a PCA across chicks of all ages to estimate body condition throughout the chick period (see Prediction 3).

In analyses of morphological data, the first principal component (PC1) can be used as an index of body size; the loading values of this principal component are all the same sign and indicate overall growth (Burnaby, 1966; Gould, 1966; Huxley, 1932; Klingenberg, 1996). In other words, small chicks are smaller than large chicks in all skeletal features. Higher‐level principal components are driven by variance in the ratios of skeletal features, after overall size is considered. Variance explained by principal components two through four (PC2, PC3, PC4) therefore provide an indication of how chicks vary in their shape (Jolicoeur & Mosimann, 1960; Klingenberg, 2016). For example, chicks with a positive score for PC2 will have relatively large features with a positive loading value for that component and relatively small features with a negative loading value for that component.

We used the broken‐stick model as the threshold for principal component significance (Figure 1b). The broken‐stick model recommends retention of principal components that explain a greater variance than the variance explained by random eigenvalues generated for same‐length vector (MacArthur, 1957). Based on this model, PC2, PC3, and PC4 (the “shape” components) should explain at least 27.1%, 14.6%, and 6.25% of variance in the skeletal features, respectively, across chicks to be descriptive of these data. We regressed the second principal component (PC2) on log‐transformed mass to determine whether, in addition to overall size, mass influences the ratio of skeletal feature sizes in this species.

### 2.6 Prediction 3: Energetic trade‐offs

To better understand how resources were allocated to skeletal features and mass, we examined the rate of change in average values of these features at each age of growth. We were also interested in body size‐specific mass as an indicator of how mass reserves varied across the chick period. We regressed chick log‐transformed mass on chick body size, measured as the first principal component for the PCA across chick ages, and used the residuals from this regression as an indicator of body condition (Figure 1c). We ran this PCA on a randomly selected subsample of our dataset that included only one set of measurements per chick to avoid repeated measures (*n* = 9,493 observations).

If Magellanic chicks of the same age vary in how they allocate their energy based on their resource availability, we would expect the log–log relationships between skeletal features and mass to be nonlinear for chicks of a given age (Figure 1c). When chicks are prioritizing mass, as we predicted for chicks fewer than 10 days of age, relatively light chicks will have relatively low skeletal feature to mass ratios (Figure 1c). However, when chicks are prioritizing feature size, as we predicted for older chicks, relatively light chicks will continue to grow skeletally rather than storing mass and will have relatively high skeletal feature to mass ratios (Figure 1c). In either scenario, the heaviest chicks of each age may reach the physiological limits of growth and place “leftover” energy into reserves and therefore have relatively low skeletal feature to mass ratios (Figure 1c).

For each age of chick growth (age 0 days to age 100 days), we tested for nonlinearity in the log–log relationship between skeletal features and mass by comparing the fit of three models: a linear model, a third‐order polynomial model, and a generalized additive model (GAM). If all chicks allocate energy similarly, a linear model should be the most parsimonious because it has fewer parameters than a GAM or polynomial model given the same set of predictor variable(s). We chose a third‐order polynomial model based on the shapes of the relationships we predicted in Figure 1 and a GAM to allow for greater flexibility in the shape of this relationship. Our GAM used cubic splines for smoothing and we set the basis parameter “*k*” to 10 based on exploratory analysis of the maximum estimated degrees of freedom across ages and features. The model with the lowest AIC was considered the best‐supported model and models that had AIC values within 2 of each other were considered to have a similar fit. When nonlinear models had the best fit, we visually examined these relationships to determine whether they suggested prioritization of skeletal growth or mass.

## 3 RESULTS

### 3.1 Prediction 1: Average patterns of growth

**FIGURE 2:**
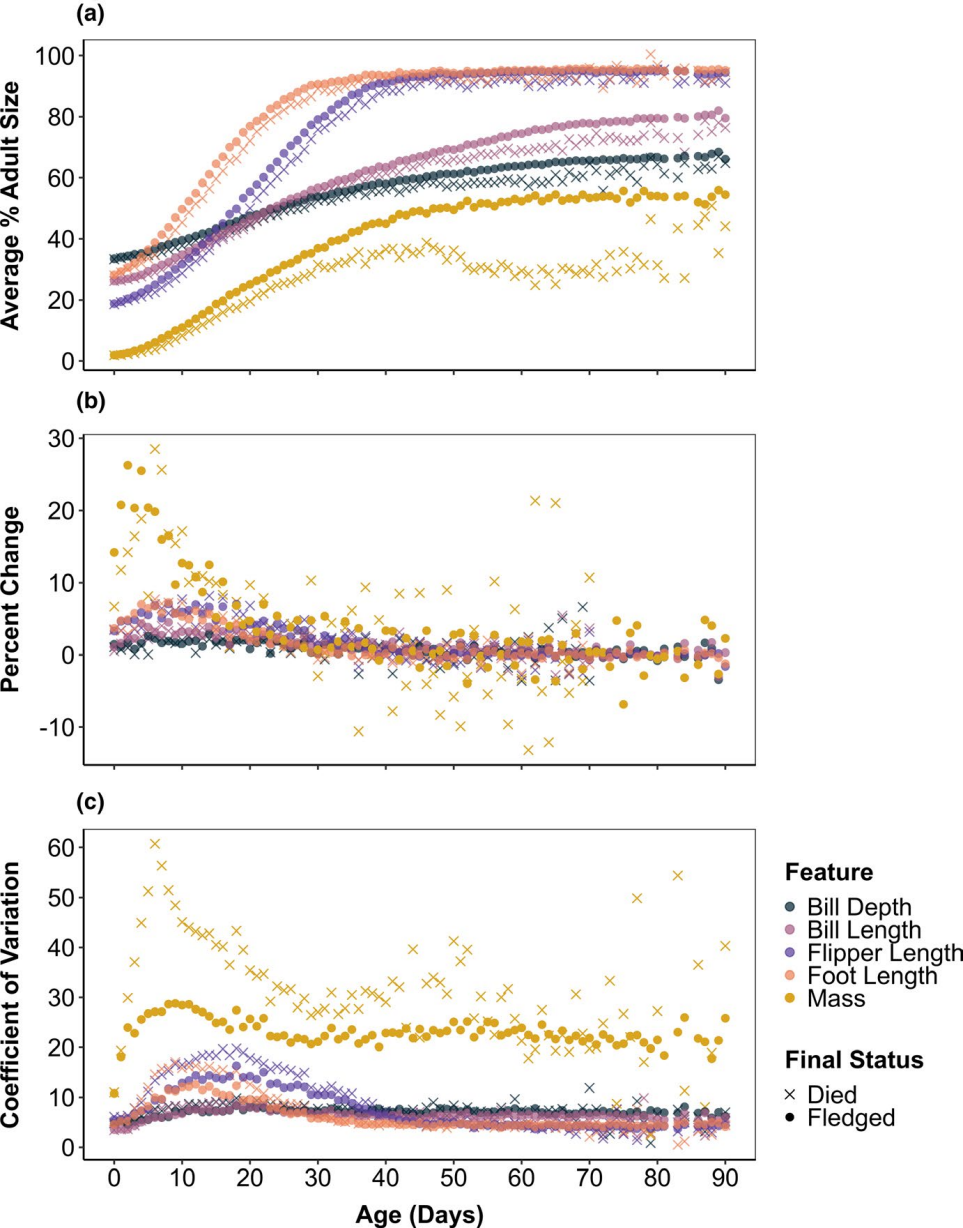
To describe average patterns of and variation in the growth of Magellanic penguin chicks at Punta Tombo, Argentina, we used data of four skeletal features (foot length, flipper length, bill length, and bill depth in cm) and mass (in kg) measured for 9,491 known‐aged Magellanic penguin chicks from 1983 to 2017. Chicks were measured when they hatched then every 5–10 days until they died or fledged. We compared these trends for chicks that eventually fledged (closed circles), defined as chicks weighing 1.8 kg on or after 10 January, and those that did not fledge (x symbols) to better understand selection on skeletal traits and mass. We assumed that chicks that were not marked as fledged eventually died, whether they were given an official death status or not. To examine how skeletal features were prioritized temporally, we calculated the size of each feature at hatching and fledging and the speed at which each feature reached 90% of the average adult male size in this species (a). We also examined how rapidly each feature was changing over the chick period, which we quantified using the percent change in the average value of that feature from one age to the next (b). For visualization purposes, percent change in mass is only shown for chicks that died through age 70 days; after this age, the percent change among these chicks became highly variable. To understand selection on and canalization of these features, we used trends in the coefficient of variation of skeletal features and mass for chicks that fledged and those that died (c)

Among chicks that fledged, bill depth (33.4 ± 1.6%) was the closest to adult size at hatching, followed by foot length (28.2 ± 1.3%), bill length (26.2 ± 0.9%), and flipper length (18.8 ± 1.0%; Figure 2a). Sizes at hatching were similar for chicks that eventually died. Bill length and depth show slower and steadier growth than foot or flipper length throughout the chick period (Figure 2b). Among chicks that fledged, the growth of bill length peaked at 8 days (4.54% increase), bill depth peaked at 14 days (2.93% increase), flipper length peaked at 8 days (7.04% increase), and foot length peaked at 8 days (7.25% increase). Foot length was the fastest growing feature through age 8 days and flipper length was the fastest growing feature from ages 9 to 37 days. In chicks older than 37 days, bills grew faster than flippers or feet (Figure 2b).

For chicks that did not fledge, the percent change in feature size from one age to the next was more variable. Because sample sizes for chicks that eventually died were lower than 50 individuals at ages greater than 50 days, here we describe peaks at ages of up to 50 days. Among these chicks, growth in bill length peaked at 7 days (4.04% increase), bill depth peaked at 24 days (2.59% increase), flipper length peaked at 14 days (8.18% increase), and foot length peaked at 6 days (7.70% increase). Therefore, as compared to chicks that fledged, bill length had an earlier and lower peak, bill depth had a later and lower peak, flipper length had a later and higher peak, and foot length had an earlier and higher peak.

On average, foot length reached 90% of adult size by age 29 days across chicks that fledged and by age 34 days among those that did not (Figure 2a), while flipper length reached 90% of adult size by age 38 days among chicks that fledged and by age 43 days among those that did not (Figure 2a). Neither average bill depth nor average bill length reached 90% of adult size prior to fledging. Chicks that fledged had larger bills at age 70 days than chicks that later died. The time it took chicks to reach 90% of the average adult size was variable; the age range was 19 to 99 days of age for foot length and 26 to 96 days of age for flipper length. When we compared measurements from remeasured chicks to their adult sizes rather than to that of the average male, chicks reached 90% of adult size at an earlier age (28 days for foot length and 34 days for flipper length) but in the same order across features.

### 3.2 Prediction 2: Variation among chicks

Foot length, flipper length, and bill depth had similar coefficients of variation (CV) at hatching (Figure 2c). The CV of foot and flipper length increased during the rapid growth phase of these features, then decreased and remained low among chicks that fledged and those that did not (Figure 2c). For bill depth and length, the CV was lower than that of foot and flipper length when chicks were young but, because bills grew throughout the chick period, was higher at fledging (Figure 2c). For chicks that eventually died, the CV of skeletal features was higher than those that fledged, particularly for flipper length and foot length during the first 25 days of growth (Figure 2c).

The CV of mass was higher than the CV of skeletal measurements throughout the chick period (Figure 2c). Mass also showed a higher CV for chicks that died than for those that fledged, particularly in young chicks; the greatest difference occurred at 6 days of age, when the CV of mass was 60.7% among chicks that eventually died and 27.11% among those that eventually fledged (Figure 2c). Among chicks that later died, the CV of mass showed an increasing trend again at ages 40 through 50 days. No such increase in the CV of mass was apparent among chicks that fledged (Figure 2c).

The first principal component (PC1), or overall size, described over 39% of the variance in skeletal feature size at all ages. As young chicks rapidly grew their skeletal features, variance due to size (PC1) increased and variance due to shape (PC2, PC3, and PC4) decreased. At most ages nine through 30 days, PC1 described over 80% of the variance in feature size across chicks at that age; it peaked at 86.5% of the variance at age 18 days. The variance described by shape began to slowly increase after this period of rapid growth, describing 33.4% of the variance (14.6% by PC2, 10% by PC1, 8.8% by PC3) among features of chicks that were 70 days of age.

The only time that variance due to higher‐order components exceeded the broken‐stick threshold was at hatching (27.1% threshold for PC2, 28.0% variance explained by PC2). In chicks zero to 4 days of age and in most chicks 35 days or older, PC2 described trade‐offs between flipper and foot length and bill size. Flipper length and foot length had positive loading values while bill depth and bill length had negative loading values. When we regressed chicks' PC2 scores on their mass at each age, we found the following significant relationships: a negative correlation at age 0 days, a positive correlation at most ages 4 through 18 days and at 24, 31, and 33 days, and a negative correlation at most ages above 37 days. Therefore, in just‐hatched chicks and in chicks older than 37 days, a greater mass was associated with longer and deeper bills relative to flipper and foot length.

### 3.3 Prediction 3: Energetic trade‐offs

**FIGURE 3:**
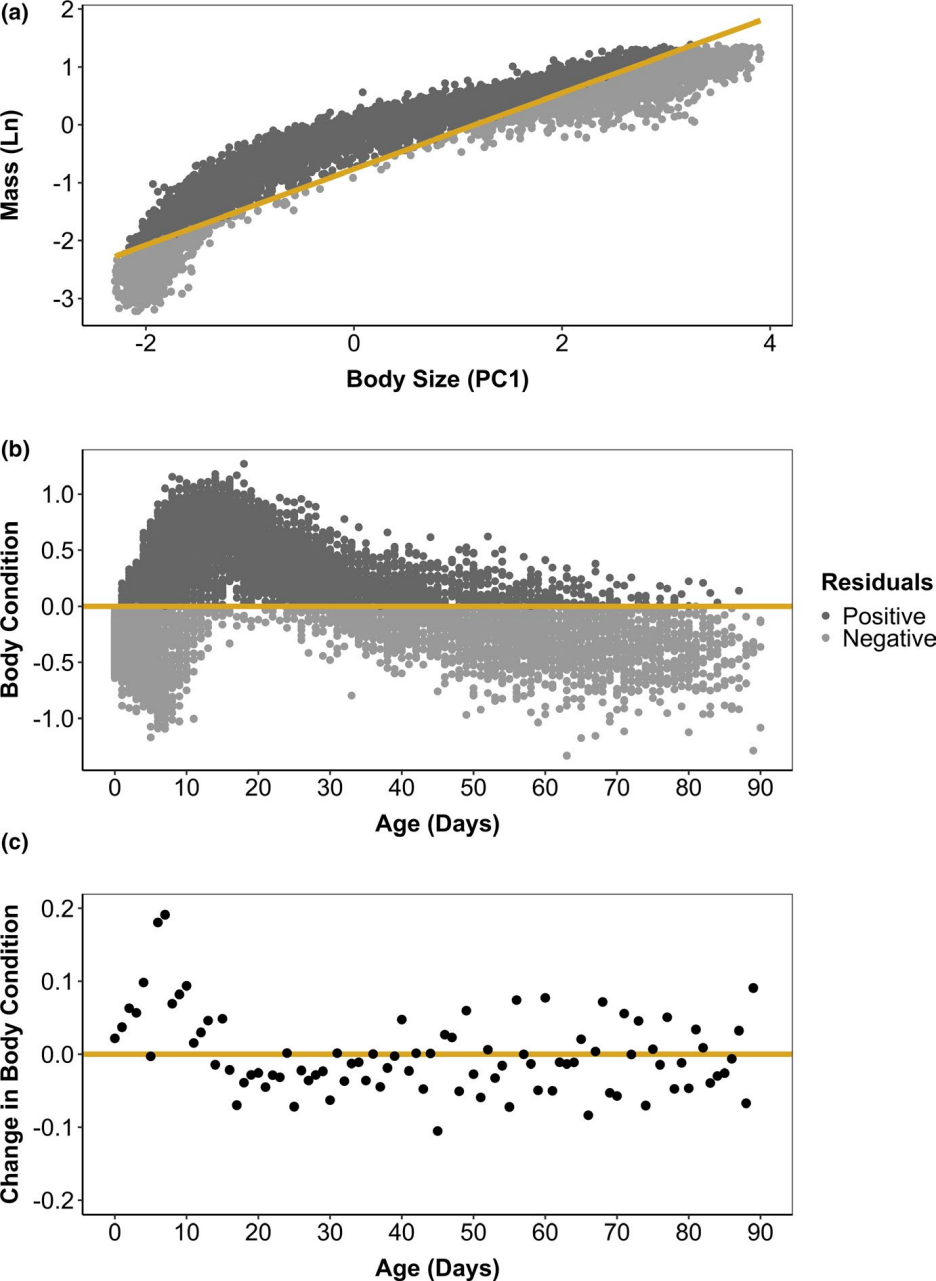
To examine how mass storage changes throughout the Magellanic penguin chick period, we regressed natural log‐transformed mass on overall body size (a) then used the residuals of this relationship to indicate body condition (b). Chicks with positive residuals have a relatively high mass for their size (high body condition), while chicks with negative residuals have a relatively low mass for their size (low body condition). Overall body size was represented by the first principal component of a principal component analysis of four skeletal features (foot length, flipper length, bill length, bill depth) measured for 9,491 Magellanic penguin chicks at Punta Tombo, Argentina, from 1983 to 2017. We measured (in cm) and weighed (in kg) chicks at hatching then every 5–10 days until they died or fledged and included one randomly chosen set of measurements from each chick in the analysis to avoid repeated measures. Body condition increased rapidly after chicks hatched and peaked early in growth, then declined throughout the remainder of the chick period (b). We also calculated how much the average body condition across chicks changes from one age to the next (c). Chick body condition increased most rapidly between 6 and 7 days and 7 and 8 days of age

Across chicks of all ages, PC1 described 97.3% of the variance in skeletal feature size. Chick mass and skeletal size were strongly correlated (*r* ^2^ = 0.89, *b* = 0.66, *p* < .0001), but this relationship was nonlinear (Figure 3a). Chick body condition, or residuals from the regression of mass on body size, was negative at hatching but rapidly increased during the first 7 days of age (Figure 3b,c). The most rapid increase in body condition occurred from ages six to seven (+0.18) and ages seven to eight (+0.19) and the most rapid decrease occurred from ages 45 to 46 (−0.11). The average body condition value peaked at 0.66 across chicks aged 16 days (Figure 1b). Body condition declined in chicks aged 16 through 39 days, then showed more variable change but a slow overall decline until the average fledging age (Figure 3b,c).

At most ages, the relationship between skeletal size (individual features and PC1) and mass was described equally well by a linear, polynomial, or generalized additive model, that is, the AIC values for these three models were within two of each other. In young chicks, however, GAMs often better described the allometric relationship between features and mass than did linear or polynomial models. Across ages and features, a linear model never performed better than a polynomial model and a polynomial model never performed better than a generalized additive model.

For overall body size, GAMs better described the relationship with mass than did linear models at all but one age for chicks aged zero through 30; GAMs provided a better fit than did polynomial models at most but not all of these ages. The difference in AIC was greatest for chicks that were 7 days of age, when AIC values were 383.1, 132.0, and 81.0 for the linear, polynomial, and generalized additive models, respectively. The results for individual skeletal features were similar. Through age 30 days, GAMs best described allometric relationships for 28 days for foot length, 28 days for flipper length, 25 days for bill length, 25 days for bill depth. Differences in AIC among models peaked between ages six through nine for foot length, flipper length, bill length, and bill depth.

The size of skeletal features and PC1 scores were significantly correlated with mass across chicks of each age (*p* < .0001), based on the smoothed‐term *p*‐value estimated for GAMs by mgcv. Mass described relatively little of the variance in any skeletal features at hatching but was most closely associated with foot length (*r* ^2^ = 0.084) and overall body size, or PC1 (*r* ^2^ = 0.10; Figure A1).

In young chicks, the skeletal feature that was most closely associated with mass was foot length. After 16 days of age, flipper length became more closely associated with mass. The highest *r*‐squared value across all features and ages was for the relationship between foot length and mass of chicks that were 7 days old (*r* ^2^ = 0.79). At ages greater than 43 days, bill length and bill depth showed a stronger association with mass than did flipper length or foot length (Figure A1). However, by fledging, little of the variation in skeletal feature size was described by mass (Figure A1).

We examined the output of GAMs visually to describe the shape of these nonlinear allometric relationships (Figure A2). Though the exact nonlinear pattern captured by the GAM varied by feature and age, this nonlinearity was most apparent for chicks that were light for their age. When the relationship was nonlinear, light chicks had a larger skeletal size for their mass than that predicted by the linear relationship, indicated by the upward “tail” in the model fits (Figure A2). The shape of the relationship predicted by third‐order polynomial models was similar to that of GAMs. Depending on age and feature, the heaviest chicks at each age sometimes had a smaller skeletal size, a larger skeletal size, or a skeletal size about equal to that predicted based on a linear relationship with mass (Figure A2). This pattern was therefore less consistent than the pattern we observed for the lightest chicks of each age.

## 4 DISCUSSION

Growing chicks must prioritize the growth of features needed to survive at each age and prepare to fledge while maintaining sufficient mass to avoid starvation. How chicks allocate their energy is driven by the amount and predictability of their resources, the strength of other pressures they face (e.g., predation pressure: Cheng, 2008), and the conditions under which they fledge. We found evidence for strong selection on the trajectory of growth in Magellanic penguin chicks based on the timing of energetic constraints and other selective pressures throughout the chick period. Though the speed of growth and time it takes to fledge varies substantially in this species, variation in the size of skeletal features and in the relative size of these features (i.e., shape) was low in fledging chicks, indicating strong selection on and canalization of these features during growth. Growth was slower and more variable among chicks that eventually died than among those that eventually fledged.

### 4.1 Prediction 1: Average growth trajectory

We found that Magellanic penguin chicks' growth trajectory closely matched the order we predicted based on the need for functionality of these features, with feet being most important during early life stages and flippers and bill becoming more important at fledging. Among Magellanic penguin chicks that fledged, feet reached adult length first, by the age (29 days) that chicks are increasingly left unguarded by parents. Large and presumably strong feet may facilitate a chick's ability to get food from parents and, when they are no longer guarded, to escape intruders or predators by moving to a nearby nest. Among chicks that fledged, flippers began growing more rapidly than feet at 9 days and reached adult length by age 38 days. Prioritization of feet early in ontogeny followed by prioritization of flippers or wings has been shown for other seabirds that rely on their feet for locomotion as chicks and flippers or wings for locomotion after fledging (Adelie penguin *Pygoscelis adeliae*, chinstrap penguin *Pygoscelis antarcticus*, gentoo penguins *Pygoscelis papua*: Volkman & Trivelpiece, 1980; California gull: Carrier & Leon, 1990; yellow‐eyed penguin: Heezik & Davis, 1990; king penguin *Aptenodytes patagonicus*: van Heezik et al., 1993; little penguin *Eudyptula minor*: Wienecke et al., 2000; common murre *Uria aalge*: Benowitz‐Fredericks et al., 2006; mallard *Anas platyrhynchos*: Dial & Carrier, 2012).

Though chicks do not need flippers for mobility until fledging, studies suggest that growth of wing components must be started early in birds (Dial & Carrier, 2012). Across 25 families of birds, species with longer wings have longer fledging periods, highlighting this constraint (Carrier & Auriemma, 1992). For Magellanic penguins, in addition to lengthening, flippers must also widen and stiffen to allow for successful hunting after fledging, and these changes do not occur until flippers have reached adult length (Boersma, Personal communication). Magellanic penguin chicks also flap their flippers vigorously weeks before fledging, presumably to build the pectoral muscle strength necessary to swim long distances (Boersma Personal communication).

### 4.2 Prediction 2: Variation among chicks

In addition to describing Magellanic penguin chicks' average growth pattern in the context of their natural history, we tested whether chicks ever varied from this pattern. Our results indicate high variation in the speed of growth across chicks. For example, while chick foot length was 90% of adult size at 29 days on average, the range of ages at which chicks reached this size was 19 to 99 days. Despite lower access to resources, chicks that eventually died did reach the same flipper and foot size as chicks that fledged, suggesting that the ultimate size of these features is constrained in this species. Their growth was slower, however. It took chicks that eventually died 5 days longer to reach 90% of the adult flipper and foot size than chicks that fledged.

The more variable a feature, the more selection can occur on this feature (Grafen, 1988) and when strong selection occurs variability should decline over time (Fisher, 1930). Our results suggest that both selective mortality and canalization reduce variation in the size of skeletal features across the chick period. The skeletal features that were prioritized early in chick growth, feet and flippers, also showed high variability. Furthermore, chicks that eventually died were more variable in these features than those that eventually fledged, indicating strong selective mortality. These features also showed canalization; variation rapidly declined as the features became fully grown and were low (less than 5%) in older chicks whether they eventually fledged or died. The remaining variation in feature size at fledging is likely to be driven in part by sexual size dimorphism.

Bill size was similarly most variable in young chicks. Variability in this feature was much lower than that of flippers or feet, however, and this variability was only slightly higher in chicks that eventually died than in those that eventually fledged. At fledging, variation in bill size was low (~6%–8%) but higher than for flippers or feet, likely due to postfledging growth of and strong sexual size dimorphism in this feature. These patterns of variation indicate weaker selection on bills than on foot length or flipper length during the chick period.

Though the size of some features was more variable than others, we found that chicks generally grew their skeletal features in similar proportions. That is, variation in feature size was largely driven by variation in overall body size rather than variation in shape. When compared to chicks that eventually fledged, foot size and flipper size both took exactly 5 days longer to reach 90% of adult size in chicks that eventually died. This finding further suggests that chicks do not alter their shape when resources are limited. They grow slower in all features but must grow their features in the same order regardless of resource availability.

Chicks were most variable in shape at hatching. The variation attributed to overall size increased during the rapid growth of young chicks, then declined slightly but remained high through the fledging period. Shape described more of the variance in feature size when the overall variance in feature size was lowest, before features started growing or after they had finished growing. Our findings suggest that, in addition to strong selection on the size of individual skeletal features, there is strong selection on shape in Magellanic penguin chicks throughout their growth period. By the time chicks fledge, there is convergence on a similar shape regardless of size.

At most chick ages, the second principal component of variation across chicks was driven by a trade‐off in the size of bills versus feet and flippers. This trade‐off was consistently present across chicks younger than 5 days and older than 34 day of age. Though it only reached the broken‐stick model cutoff in just‐hatched chicks, this variation may still be biologically meaningful.

At hatching, prehatching prioritization of foot growth may explain this variance; foot length and flipper length of just‐hatched chicks have a stronger correlation with egg size than does bill size in this species (Reid & Boersma, 1990). At fledging, variation in the ratio of bills to flippers and feet is likely the result of sexual size dimorphism. Mass shows high sexual size dimorphism and bill size is more dimorphic than flipper or foot size (Boersma et al., 2013). Accordingly, we found that heavier chicks at fledging have large bills relative to their foot and flipper length.

### 4.3 Prediction 3: Energetic trade‐offs

At Punta Tombo, the largest driver of chick mortality is starvation, and this mortality is greatest during the first 10 days after a chick hatches (Boersma & Rebstock, 2014; Boersma & Stokes, 1995). Chicks must be fed soon after hatching or their digestive tract will not properly develop and, due to their small size and low storage capacity, they will die if their parents do not bring back a meal every 1–3 days. Our results suggest that, to survive this vulnerable period, chicks prioritize mass storage immediately after hatching. Skeletal features are growing rapidly in these young chicks, but mass shows a relatively high rate of increase, leading to daily improvements in body condition throughout the first week of chick growth. This period of storing mass is also associated with very high variability in mass across chicks. Among chicks that did not survive to fledge, the coefficient of variation in mass peaked at 60.7% at 6 days then decreased rapidly through 10 days of age, indicating strong selection due to starvation of chicks without sufficient mass.

The rapid growth of skeletal features and mass during the first few days after hatching also supports the energy allocation hypothesis (Węgrzyn, 2013), as Magellanic penguin chicks begin thermoregulating at around 10 days of age (Boersma & Rebstock, 2014). At Punta Tombo, young chicks die from hypothermia following precipitation events (9–23 days of age) and from hyperthermia later in the breeding season, when temperatures at the colony can reach over 37°C (Boersma & Rebstock, 2014, Holt and Boersma, Personal communication). Thermoregulation is costly (e.g., up to 31% of metabolic rate in Adelie penguin chicks: Chappell et al., 1990; up to 35% of metabolic rate in Eurasian blackcap *Sylvia atricapilla*: Węgrzyn, 2013) and rapid growth may reduce the costs of thermoregulation by decreasing surface‐to‐volume ratio and by creating muscles that can produce heat through shivering (Cheng & Martin, 2012; Dégletagne et al., 2013; Pereyra & Morton, 2001; Stahel et al., 1987; Starck & Ricklefs, 1998). In older chicks, having large flippers, feet, and bills aids in heat loss (Starck & Ricklefs, 1998; Symonds & Tattersall, 2010; Tattersall, 2016; Whittow & Rahn, 1984).

Furthermore, we found that the completion of flipper growth coincides well with the onset of juvenile feather growth in Magellanic penguin chicks, around 40 days of age, reducing energetic conflicts between skeletal and feather growth. Growing feathers is resource‐intensive (in macaroni and rockhopper penguins: Brown, 1986), highly sensitive to diet quantity and quality (in African penguins *Spheniscus demersus*: Heath & Randall, 1985; in common murre: Benowitz‐Fredericks et al., 2006), and a necessary prerequisite for fledging (Boersma, Personal communication). Completing flipper and foot growth early on reduces energy conflicts as chicks prepare to fledge.

A temporal mismatch in energy spent on feathers and energy gained from parents may explain high starvation rates in chicks aged 40 to 60 days (Boersma & Stokes, 1995). At most ages during this period, chick body condition is low and declining. Among chicks that did not survive to fledge, variability in mass increased from age 40 to 50 days, then declined, indicating a second period of strong selection on mass; this change in variability was not seen in chicks that eventually fledged.

As predicted, the relationship between skeletal features and mass was nonlinear in young chicks, a period when mass, flippers, and feet were rapidly growing. Based on visual inspection, however, the shape of this relationship did not match our predictions. We expected young chicks that were light for their age to prioritize mass and therefore have relatively low skeletal size to mass ratios, but these chicks had relatively high skeletal size to mass ratios. There are two possible and nonmutually exclusive explanations for this pattern: (a) chicks that are relatively light for their age grew rapidly when they had sufficient resources but subsequently did not get fed and lost mass, leading to high ratios of skeletal size to mass among these chicks and (b) chicks need to maintain some skeletal growth, even when the risk of starvation is high. In common murres, for example, chicks switch to increased allocation to wing elements around the same age (15–20 days) regardless of diet and mass (Benowitz‐Fredericks et al., 2006). This switch suggests that prioritization of skeletal features is ontogenetically determined in some seabirds rather than resource‐dependent.

Relationships between skeletal size and mass continued to show high skeletal size to mass ratios for light chicks through age 30 days. At 11–30 days of age, past the period of peak starvation, these patterns are in line with our prediction of skeletal prioritization in older chicks. After age 30 days, the relationship was well supported by either a linear or nonlinear model, but the strength of the relationships between mass and skeletal features declined and was low at fledging. Therefore, although there is strong canalization of skeletal shape, that is, the ratio of skeletal features to each other, this canalization does not seem to be present for the ratio of mass to skeletal size.

Though perhaps unsurprising for some features (e.g., foot length), we would expect the ratio of flipper length to mass to be somewhat constrained (Sullivan et al., 2019). However, only 15% of the variation in flipper length was associated with variation in mass at fledging. We did not find evidence that chicks lose mass in preparation to fledge as is common in some other penguin species; Adelie penguin chicks, for example, lose 10%–15% of their peak mass before they fledge (Ainley et al., 2018). In contrast, many Magellanic chicks appear to wait until they receive a substantial meal before fledging (Boersma personal obs.)

Among the best‐fed chicks, we predicted that mass would increase without skeletal growth due to physiological limits on this growth. Though the relationship between skeletal features and mass was nonlinear in chicks through age 30 days, we did not find consistent evidence that the heaviest chicks of a given age had reached physiological growth limits, that is, the skeletal feature to mass ratio did not decrease in the heaviest chicks. This result suggests that food availability remains the limiting factor to growth even among the best‐fed chicks of a given age. High rates of feeding may also signal to chicks that resource availability is high, reducing the need to store mass. In Adelie penguin chicks, for example, high food provisioning rates lead to lower body condition at fledging, suggesting that high food availability prompts prioritization of skeletal growth (Whitehead et al., 2015).

## 5 CONCLUSIONS

Magellanic penguin chicks show adaptive growth. The temporal patterns in their energy allocation are closely linked to their natural history: chicks store mass when the risk of starvation is highest and grow skeletal features in the order that they are needed to survive: feet first, then flippers, and finally bills. They also minimize energy use conflicts by growing rapidly prior to thermoregulation and by completing most of their skeletal feature growth before growing their juvenile feathers. We found evidence that these fine‐tuned behaviors result from strong selective mortality throughout the chick period and that there is canalization of skeletal features during this period. As a result, variation in these features and in the relative size of these features, or the skeletal shape of chicks, is low at fledging.

## CONFLICT OF INTEREST

The authors have no conflicts of interest to declare.

## AUTHOR CONTRIBUTION

**Natasha Jeanne Gownaris:** Conceptualization (equal); Formal analysis (lead); Investigation (supporting); Visualization (lead); Writing‐original draft (lead); Writing‐review & editing (lead). **P. Dee Boersma:** Conceptualization (equal); Funding acquisition (lead); Investigation (lead); Writing‐review & editing (supporting).

## ETHICAL APPROVAL

We acquired animal use permits from the University of Washington (IACUC 2213‐02) and research permits from the Province of Chubut and the Offices of Tourism and of Flora and Fauna for each year of the study.

## Supporting information

FigA1

FigA2

AppendixS1

Supinfo

## Data Availability

All age‐aggregated data associated with this manuscript are available on Dryad Digital Repository along with a README file (https://doi.org/10.5061/dryad.t76hdr80g). One data file (Statistics_ByFeatureAge.txt) includes descriptive statistics for all the features we measured and key outputs for each age‐specific model (linear model, generalized additive model, third‐order polynomial model). The other data file (PCAOutputs_ByAge.txt) includes age‐specific information on the variance explained by the first principal component (overall size) and higher‐order principal components (shape) at each age and on the loading values of skeletal features for the first and second principal components.
