# Supplementary figures and images for: Feet first: Adaptive growth in magellanic penguin chicks
Source: Ecol Evol. 2021 Mar 13;11(9):4339–52. doi: 10.1002/ece3.7331 (PMC8093740; doi:10.1002/ece3.7331)

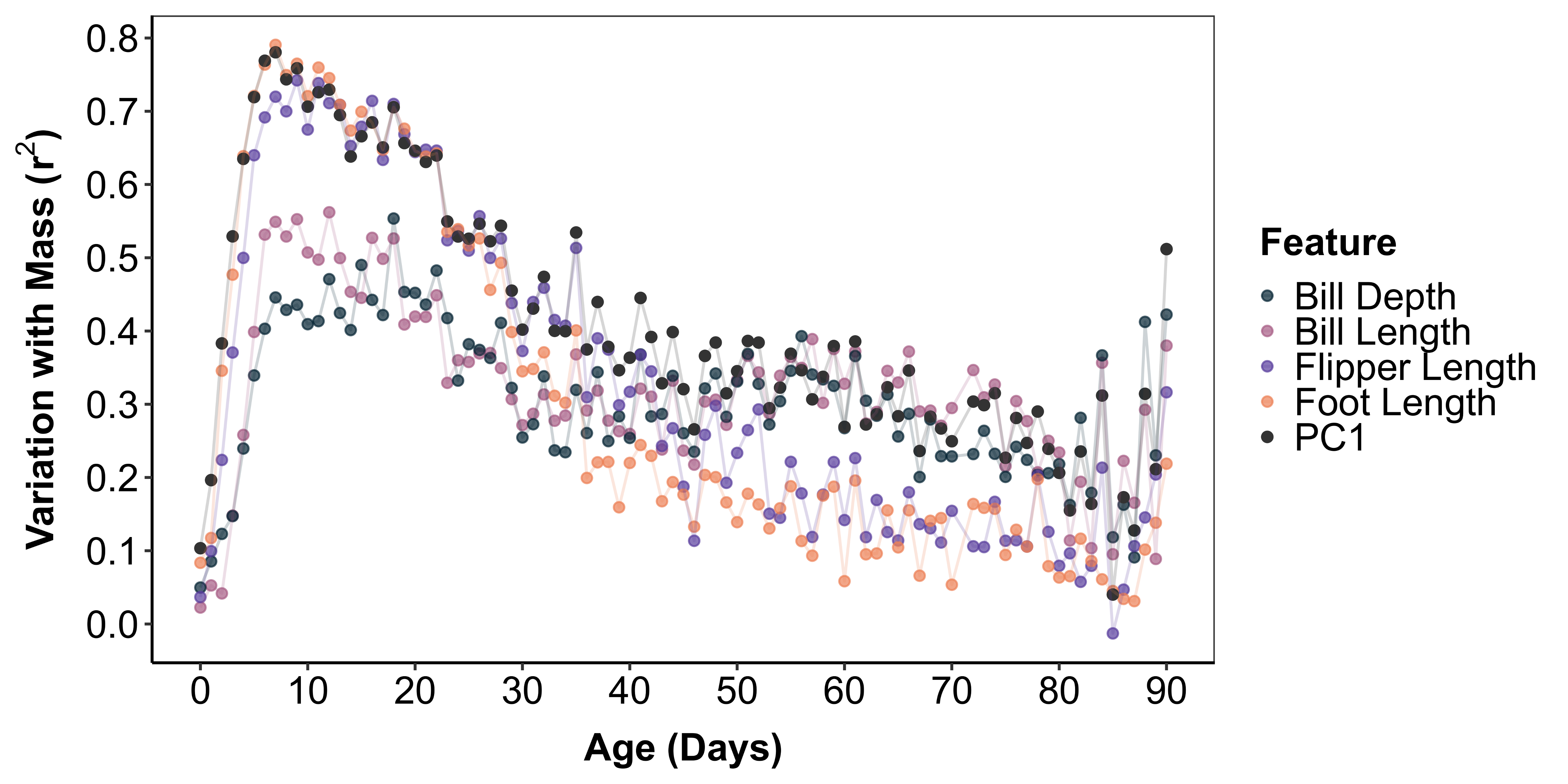

Supplement: Supplementary file 1 — FigA1 [file ECE3-11-4339-s003.tiff]

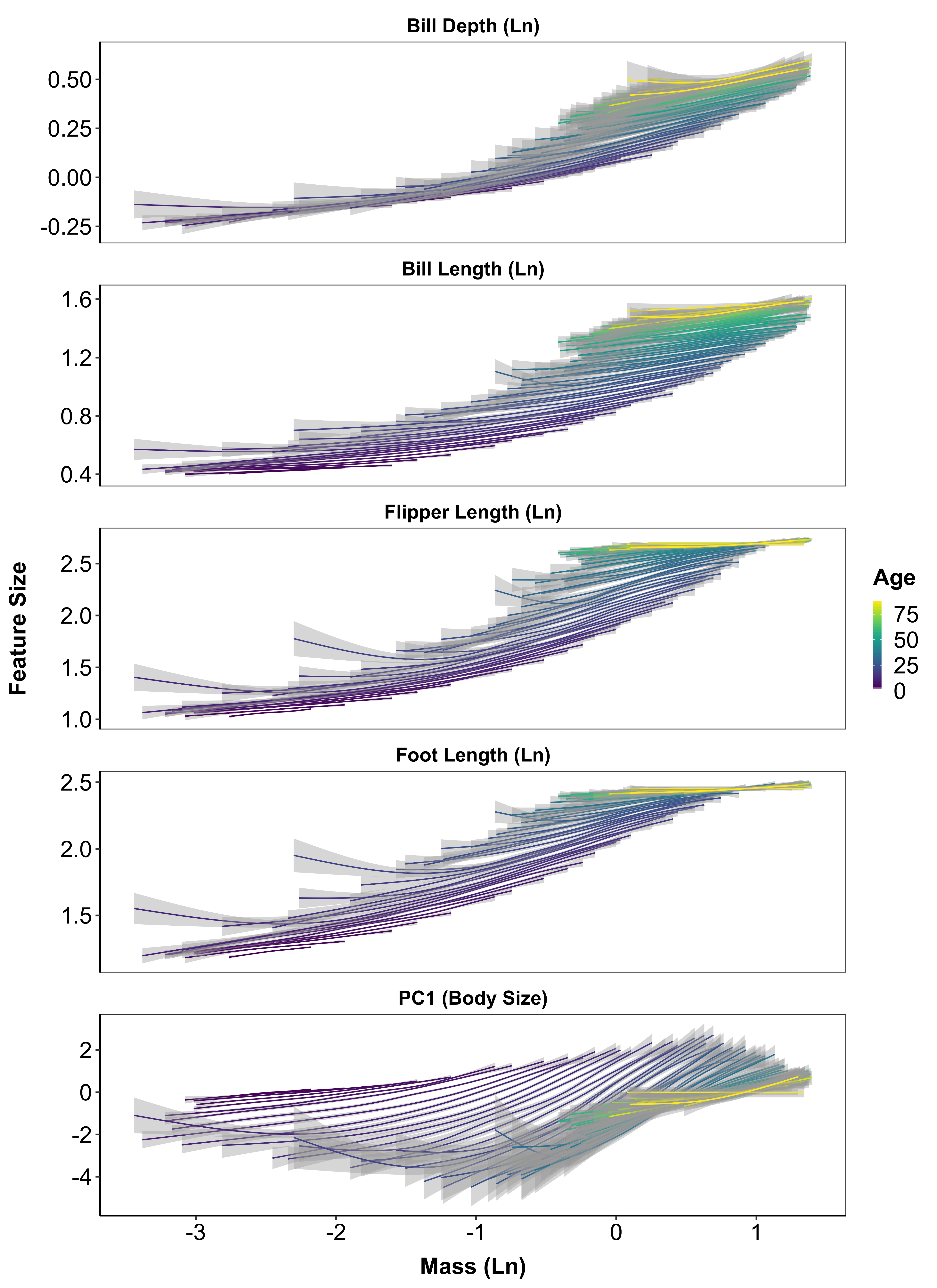

Supplement: Supplementary file 2 — FigA2 [file ECE3-11-4339-s001.tiff]
