# Supplementary material for: Feet first: Adaptive growth in magellanic penguin chicks
Source: Ecol Evol. 2021 Mar 13;11(9):4339–52. doi: 10.1002/ece3.7331 (PMC8093740; doi:10.1002/ece3.7331)
Supplement: Supplementary file 3 — AppendixS1 [file ECE3-11-4339-s002.docx]

**Appendix 1: Data Collection**

Over the course of this long-term study, we collected data on skeletal measurements and mass for 12,365 known-aged chicks from a total of 3,404 nests. Each nest was sampled anywhere between one and 17 years (2.18 ± 1.81 [x̅±SD]). We only included chicks for which we were certain of hatch date within one day, i.e., in nests visited at least one to several days before hatching and on the day the chick hatched. Over the course of the study, in nearly all nests (92.13%) where at least one chick hatched, both chicks hatched. Therefore, most of the chicks in this study (89.7%) were in nests that initially had two chicks.

We measured mass using spring scales with precision dependent on the size of the chick (from 1 g precision for chicks weighing less than 100 g to 50 g precision for chicks weighing over 1 kg). We measured bill length and bill depth using stainless steel dial calipers. We measured flipper length (elbow joint to the tip of the flipper) and foot length (heel to the tip of the middle toenail) of small chicks using a stainless-steel dial caliper and of large chicks (foot greater than 5 cm) using a ruler with an accuracy of 1 mm.

All chicks were marked and measured on the day that they hatched and thereafter on a five- to ten-day cycle, depending on year and nest area, until they died (n = 5,695), fledged (n = 4,535), or went missing before meeting fledging criteria (n = 2,135). Measurement cycles are based on nest area and not on specific hatch dates, so our data span the entire chick period with an average sample size of 672±220 measurements per day of growth from one to 70 days post-hatching; we measured 10,953 chicks on the day that they hatched.

Because of our interest in allometric relationships, we only included measurement occasions that included all five trait measurements: mass, bill depth, bill length, foot length, flipper length. This led to a total of 62,363 measurements of each skeletal feature and mass. Fledging occurs around 70 days of age, so our sample size for measurements begins to decline rapidly when chicks are more than 70 days of age. We did not include measurements taken when a chick was found dead. The average number of measurements per chick was 5.04±3.18, the mode was two measurements, and the maximum was 20 measurements. We identified outliers for each age and feature (>2.5 median absolute deviations) and excluded measurement occasions with outliers from our analysis, leading to a final sample size of 57,645 measurements for each feature. Exploration of our data suggested that, while some outliers were biologically realistic (i.e., a very small foot on a chick hatched at only 45 g), most resulted from recording errors.

When feet were approximately 9 cm in length, we tagged chicks with a small tag on the inner webbing of their foot. To identify first- and second-hatched chicks prior to this size, we marked first-hatched chicks with a blue marker on their left side and second-hatched chicks with a green marker on their right side. When we did not color chicks, we attached fiber tape bands on the left flipper for first hatchlings and on the right flipper for second hatchlings with identifying information of nest and chick number. We removed fiber bands and applied a new band the next time the chick was measured. Chicks that survived past January 10^th^ and weighed at least 1,800 grams were recorded as fledged unless they were later found dead (Boersma et al. 1990). We banded chicks before they fledged with a stainless-steel band on the left flipper (Boersma and Rebstock 2010).
