# Supplementary material for: Feet first: Adaptive growth in magellanic penguin chicks
Source: Ecol Evol. 2021 Mar 13;11(9):4339–52. doi: 10.1002/ece3.7331 (PMC8093740; doi:10.1002/ece3.7331)
Supplement: Supplementary file 4 — Supinfo [file ECE3-11-4339-s004.docx]

Figure A1 We measured four skeletal features in cm (foot length, flipper length, bill length, bill depth) and of mass in kg throughout the chick period for 9,491 know-aged Magellanic penguin chicks at Punta Tombo, Argentina from 1983 to 2017. We tested whether allometric relationships between log-transformed skeletal featured and log-transformed mass were nonlinear by comparing linear, polynomial, and generalized additive model fits based on Akaike's information criterion. Skeletal features changed nonlinearly with mass, particularly in young chicks. We used the r-squared value estimated by package mgcv in R to examine trends in the variance of skeletal features described by mass across chick ages. We repeated this analysis for overall body size, measured as the first principal component (PC1) of a principal component analysis on the four skeletal features that we measured, and similarly found relationships between this variable and mass to be nonlinear and to be strongest in young, rapidly growing chicks.

Figure A2 We tested whether individuals varied in their allometry depending on their resource availability by visually examining nonlinear model fits between log-transformed skeletal features and log-transformed mass at each age of chick growth. These relationships are based on measurements for 9,491 known-aged Magellanic penguin chicks at Punta Tombo, Argentina from 1983 to 2017; at each age, log-transformed skeletal feature size (bill depth, bill length, flipper length, foot length in cm) was regressed on a smoothed term for log-transformed mass (in kg) using generalized additive models (GAMs) in the mgcv package in R. We used cubic splines for smoothing and set the basis parameter "k" to 10. Sample size ranged from 3,299 chicks at hatching to 48 chicks at age 90 days; sample size was above 300 chicks through age 70 days. We also ran this regression for overall body size, measured as the first principal component (PC1) of a principal component analysis on the four skeletal features that we measured. We predicted that among young chicks, which are particularly vulnerable to starvation, light individuals would prioritize mass so have low skeletal size to mass ratios (downward facing "tail" on the GAM fit). In older chicks, which need to be mobile, we predicted that light individuals would prioritize skeletal features so have high skeletal size to mass ratios (upward facing "tail" on the GAM fit). We also expected the heaviest chicks of all ages to have low skeletal size to mass ratios due to reaching physiological growth limits and storing extra mass.
